# Current and future options for adult biventricular assistance: a review of literature

**DOI:** 10.3389/fcvm.2023.1234516

**Published:** 2023-11-06

**Authors:** Claudia Maria Loardi, Marco Zanobini, Gabriella Ricciardi, Emmanuelle Vermes

**Affiliations:** ^1^ Department of Cardiac Surgery, Tours University Hospital, Tours, France; ^2^ Department of Cardiac Surgery, Centro Cardiologico Monzino IRCCS, Milan, Italy; ^3^ Department of Cardiac Surgery, Lille University Hospital, Lille, France; ^4^ Department of Cardiology, Amiens University Hospital, Amiens, France

**Keywords:** end-stage heart failure, biventricular assistance, total artificial heart, left ventricular assist device, orthotopic heart transplantation

## Abstract

In cardiogenic shock various short-term mechanical assistances may be employed, including an Extra Corporeal Membrane Oxygenator and other non-dischargeable devices. Once hemodynamic stabilization is achieved and the patient evolves towards a persisting biventricular dysfunction or an underlying long-standing end-stage disease is present, aside from Orthotopic Heart Transplantation, a limited number of long-term therapeutic options may be offered. So far, only the Syncardia Total Artificial Heart and the Berlin Heart EXCOR (which is not approved for adult use in the United States unlike in Europe) are available for extensive implantation. In addition to this, the strategy providing two continuous-flow Left Ventricular Assist Devices is still off-label despite its widespread use. Nevertheless, every solution ensures at best a 70% survival rate (reflecting both the severity of the condition and the limits of mechanical support) with patients suffering from heavy complications and a poor quality of life. The aim of the present paper is to summarize the features, implantation techniques, and results of current devices used for adult Biventricular Mechanical Circulatory Support, as well as a glance to future options.

## Introduction

End-stage Heart Failure (HF) represents a growing disease, responsible for about one-third of total deaths (1). Although Orthotopic Heart Transplantation (OHT) is the definitive treatment, the number of patients who die while on the waiting list is still too high. Different solutions have been proposed to expand the number of hearts available for transplantation (1): the use of Hepatitis C virus positive donors is possible thanks to the advent of direct-acting antiviral therapies, while the use of the Organ-Care-System employ for extended-criteria donors allows good short-term post-transplant survival. Moreover, donation after circulatory death is now an option. In addition, cardiac xenotransplantation from genetically multi-modified organ-source pigs is an emerging new option, as demonstrated by the consistent long-term success of heterotopic (non-life-supporting) abdominal and life-supporting orthotopic porcine heart transplantation in baboons, and by a recent “compassionate use” transplant of the heart from a genetically multi-modified pig with ten modifications into a terminally ill patient who survived for 2 months (2).

However, there remains an organ shortage, which contributes to the exponential spread of Mechanical Circulatory Support (MCS) (3). This implies that cardiologists and caregivers will increasingly be faced in their daily lives with patients implanted with MCS. Moreover, the role of physicians in understanding when optimized pharmacotherapy is no longer sufficient and, as a consequence, in deciding when to address patients for MCS or OHT screening is an issue set to grow in importance.

The majority of HF patients may be adequately treated with a Left Ventricular Assist Device (LVAD), but the small percentage affected by advanced biventricular dysfunction are candidates for long-term biventricular support. As expected, not only are biventricular HF patients a more ill population, but the use of Biventricular Mechanical Circulatory Support (BMCS) doubles the mortality rate and is associated with a higher incidence of complications (4).

## Classification

Several classifications may be used to describe biventricular MCS (Table 1).

**Table 1:** Biventricular assist devices features.

Device	Assistance length	US approval	Mechanism	Flow type	Implantation
Impella 2.5/3.5/5.0/5.5 + RP	Short-term	Yes	ED, AX	Continuous	Percutaneous surgical
Tandem Heart	Short-term	Yes	CE	Continuous	Percutaneous
CentriMag	Middle-term	Yes	ED, CE	Continuous	Percutaneous
ECMO	Short-term	Yes	ED, CE	Continuous	Percutaneous surgical
Syncardia CardioWest	Long-term	Yes	PD	Pulsatile	Surgical
Berlin Heart	Long-term	Pediatric	PD, FV	Pulsatile	Surgical
2 HeartMate 3	Long-term	Off-label	ED, CE	Continuous	Surgical
Bivacor	Long-term	Under development	ED, CE	Continuous	Surgical
CARMAT	Long-term	Under development	ED	Pulsatile	Surgical

AX, axial pump; CE, centrifugal pump; ED, electrically driven; FV, vacuum-assisted filling; PD, pneumatically driven; US, United States.

The first distinction concerns short-term and long-term devices; the former group includes both percutaneous and surgically placed non-dischargeable devices (clearly indicating that, due to the acute setting, patients cannot be discharged from the hospital and managed at home), while the latter is composed of the Total Artificial Heart (TAH) (orthotopic devices which requires excision of the heart) and heterotopic machines that support both ventricles in parallel to the native ones (Biventricular Assist Devices—BiVADs). This classification derives from the new OHT allocation 6-tiered system (5).

BIVADs may ensure a minimum flow sufficient to patient's survival in case of device failure and allow for recovery.

Other classifications are based on pumps mechanisms (pneumatic, electric, axial or centrifugal), type of flow produced (pulsatile or continuous) and on emplacement (paracorporeal or intracorporeal) (6).

## Temporary percutaneous devices

Short–term percutaneous assistance is represented by the Impella (Abiomed, Danvers, MA) system and the TandemHeart (CardiacAssist Inc, Pittsburg, PA).

**Figure 1:**
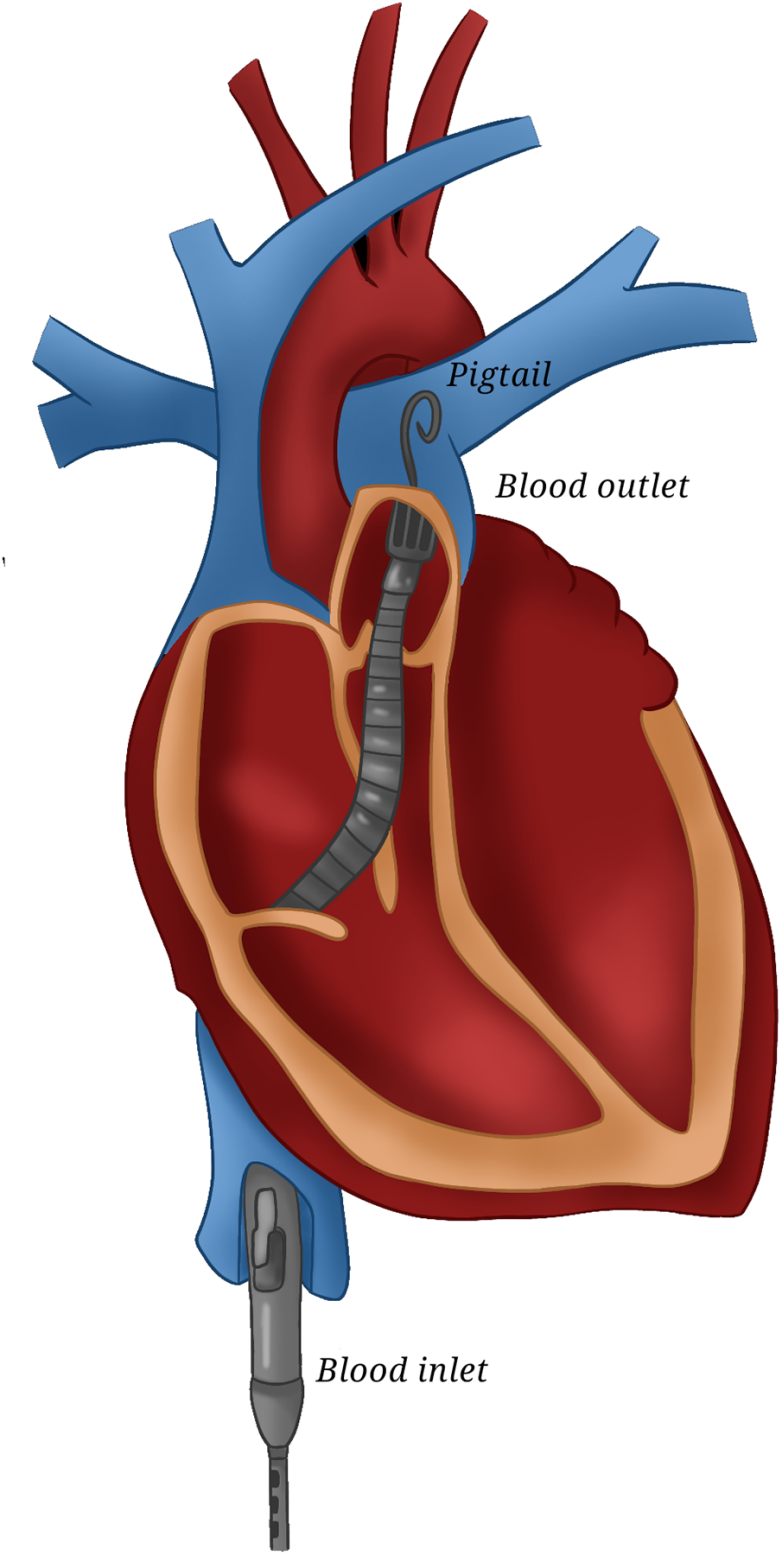
ImpellaRP. A picture showing ImpellaRP configuration.

The Impella family of products (7) includes 4 microaxial pump sizes for left ventricular support (2.5, 3.5/CP, 5.0, 5.5 L/min) and one size for RV support (RP, Figure 1) which can be coordinated with each other to produce a biventricular assistance setting (Bi-Pella). For left-sided support, the pump is inserted into a peripheral artery and positioned retrograde through the aortic valve.

Strict check and monitoring of the device position across the aortic valve with fluoroscopy and echocardiography is mandatory. The Impella 2.5, Impella CP, Impella 5.0, and Impella 5.5 can provide antegrade flow up to 2.5, 4.0, 5.0, and 5.5 L/min respectively. By continuously drawing blood from the left ventricle (LV), the Impella unloads the LV, thereby decreasing its work and myocardial oxygen demand. In addition, by delivering large volumes of blood to the aorta, Impella operation results in an increase in mean arterial pressure and cardiac output, thus improving systemic perfusion and coronary flow which augments the chances of cardiac recovery. Finally, Impella leads to a decrease in pulmonary wedge pressure and a secondary reduction in right ventricular afterload (7).

Basing on the evidence that prolonged LV unloading with LVAD promotes reverse remodeling, cardiac repair, and pulmonary vascular resistance reduction, the impact of prolonged support (until 40 days) with an LV-Impella (the PROPELLA concept) has been investigated in the context of fulminant myocarditis with encouraging results (8).

The right-sided device (Impella RP) can provide up to 4 L/min of flow. This 22F pump mounted on an 11F catheter is inserted percutaneously over a guidewire guided by fluoroscopy and/or echocardiography from the femoral vein via the right heart to the pulmonary artery, unloading the RV.

Impella pumps cannot be coupled to an oxygenator and are appropriate for 5–21 days.

**Figure 2:**
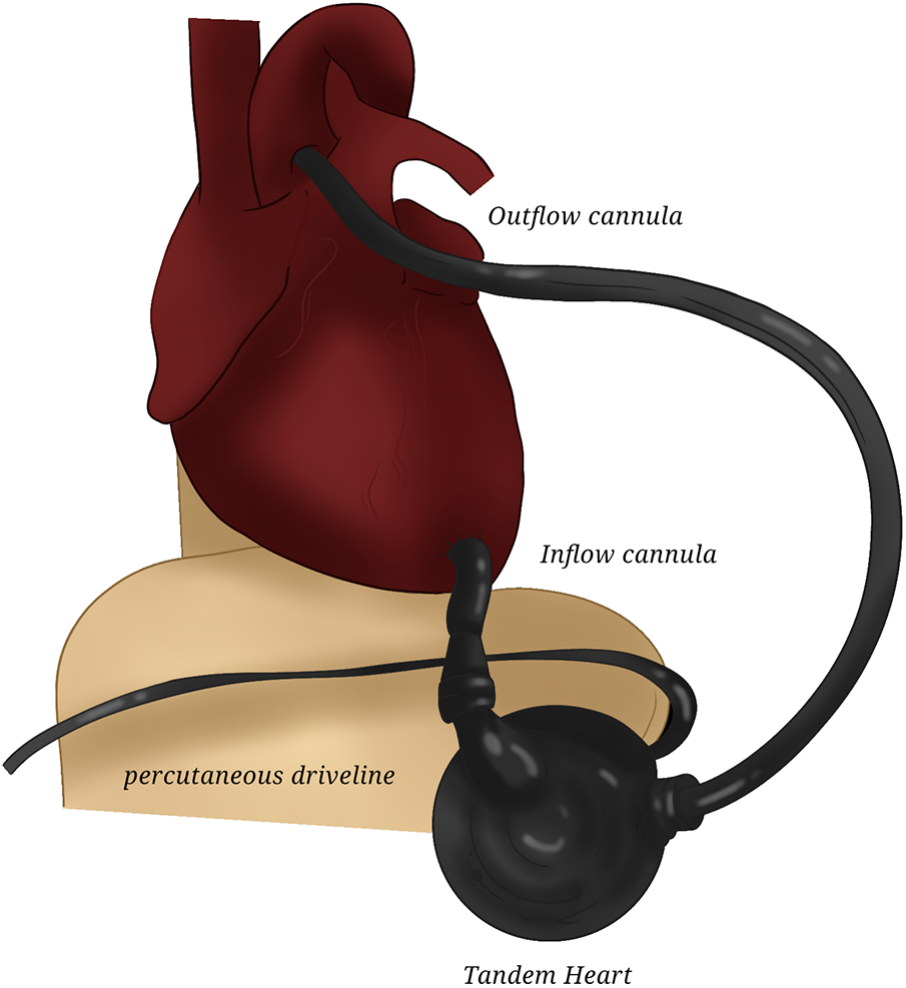
TandemHeart. The drawing represents the central placement of TandemHeart for left assistance.

TandemHeart (CardiacAssist, Pittsburgh, PA, USA) (7) represents the first totally percutaneous biventricular assist device on the market: using a 21-Fr drainage catheter inserted into the femoral vein, which traverses the right atrium into the left, a centrifugal pump, and an arterial catheter in the femoral artery, it can achieve flows of up to 5 L/min. The system can also be placed in a central cannulation setup (Figure 2).

**Figure 3:**
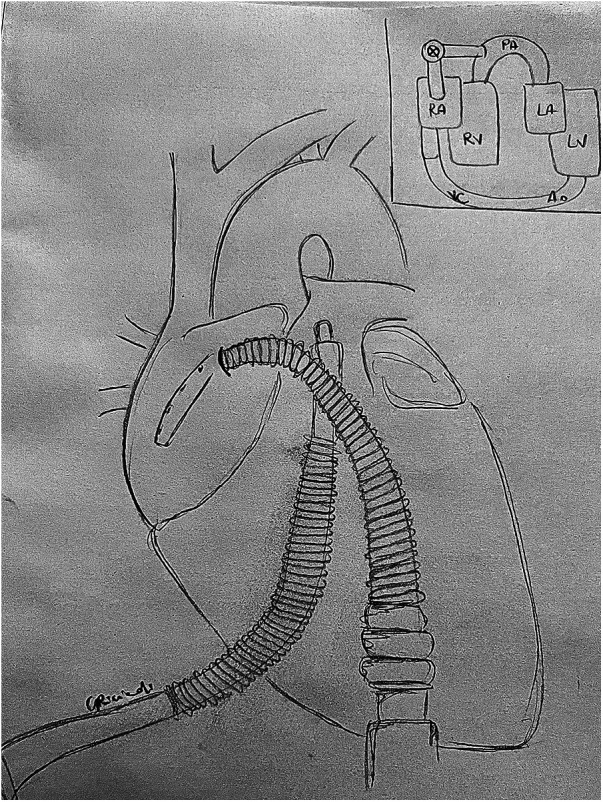
TandemHeart RVAD: central cannulation setup. An inflow cannula is placed in the right atrium and an outflow cannula in the pulmonary artery to achieve right heart assistance. LA, left atrium; LV, left ventricle; PA, pulmonary artery; RA, right atrium; RV, right ventricle.

To achieve right heart assistance, an inflow cannula is placed in the right atrium and an outflow cannula in the pulmonary artery. Both are venous cannulas, generally accessed through the left and right femoral veins, respectively, or can be surgically placed after chest opening (Figure 3). Alternatively, an internal jugular vein can be accessed to place the outflow cannula or the more recent Protek DuoR dual-lumen cannula (CardiacAssist, Pittsburgh, PA, USA): it contains two lumens, with one serving as an inflow tract positioned in the right atrium, and the other serving as the outflow tract positioned in the pulmonary artery. It is able to provide support of intermediate duration (up to weeks) with the possibility of accommodating an oxygenator.

An alternative option of biventricular assistance is represented by the association of a left Impella via the axillary artery with a Protek Duo cannula inserted in the jugular vein connected to a TandemHeart pump. Such total percutaneous configuration allows patient's mobilization with lower risk of infections while providing total heart support (9).

Depending on the basal disease, left and right ventricle may present different delay or degree of recovery: the use of two separate pumps to assist the left and the right side allows progressive and distinguished trials of weaning by monitoring the response of the two ventricles to every decrease of their respective support (8).

## Temporary surgically placed non-dischargeable biventricular devices

A number of stand-alone (without an incorporated oxygenator) centrifugal pumps which can be coupled with various cannulas are available. These are surgically inserted, using either a central or peripheral technique. CentriMag (Abbott Laboratories, Abbott Park, IL) and Rotaflow (Maquet, Rastatt, Germany) are two common examples of magnetically suspended pumps which can support of up to weeks/months in duration (7). Compared with older-generation pumps, these newer devices have less stasis and turbulent flow, allowing a partial solution to hemolysis and thrombi formation.

## Extracorporeal membrane oxygenator (ECMO)

The veno-arterial ECMO represents the most common BMCS device thanks to its simple and quick deployment: this centrifugal pump including an oxygenator/heat exchanger has traditionally been surgically implanted, but recently percutaneous cannulation has been increasingly used with a trend towards lower mortality and fewer complications (10). Available drainage cannulas size ranges from 19 to 29 Fr, whereas arterial ones range from 15 to 23 and they are chosen depending from patient's body surface area, vessels diameter and predicted needed flow support. Usually, a peripheral canulation under fluoroscopic and/or echocardiographic guidance is performed (femoral vein-femoral artery or femoral vein-axillary artery), but especially in case of post-cardiotomy failure or in the presence of significant arteriopathy, a central cannulation (right atrium-ascending aorta) is preferred.

ECMO can achieve up to 4–10 L/min flow. Maximal duration of support is very variable in relation to patient's age and basal condition: typically, haemorragic or infective complications begin to arise 10–15 days after the implantation (10).

## Post-implantation management, results, recovery, and ideal features

All these short-term BMCS require a variable degree of anticoagulation.

Table 2 summarizes their respective main characteristics.

**Table 2:** Temporary mechanical circulatory support devices.

Device	Impella 2.5–3.5	Impella 5.0–5.5	Impella RP	Tandem Heart	ECMO
Assistance length	Up to 21 days	Up to 21 days	Up to 21 days	Up to 21days	7–20 days
Implantation	Percutaneous Femoral artery	Surgical Axillary artery	Percutaneous Femoral vein	Percutaneous Femoral vein and artery Jugular vein	Percutaneous Surgical Femoral vessels Axillary artery Central
Maximal flow (L/min)	2.5–3.5	5–5.5	4	5	10
Left ventricular unloading	Yes	Yes		Yes	No
Anticoagulation	Yes	Yes	Yes	Yes	Yes

During ECMO therapy, most centers use unfractioned heparin to prevent thrombosis, with different therapeutic targets depending from the patient's procoagulant state and hepatic function, and from its tendance to spontaneously bleeding (for instance at the site of peripheric cannulas insertion or in thoracic drains in case of central surgical cannulation). However, heparin induced thrombocytopenia and heparin resistance are conditions frequently requiring the use of different anticoagulants. In this context, direct thrombin inhibitors like bivalirudin and argatroban have been established as alternatives. A recent meta-analysis (11) showed that the use of bivalirudine resulted in better clinical outcomes and in a reduced risk of thrombosis as compared to heparin.

Concerning Impella, according to the manufacturer, the goal activated clotting time (ACT) is 160–180 s: in some patients, purge heparin may be sufficient to achieve anticoagulation goals; when insufficient, the addition of titratable, supplemental non-purge heparin is required to provide optimal anticoagulation. Two different dextrose concentrations (5% and 20%) in the purge solutions have been tested with similar therapeutic activated partial thromboplastin time rates, thrombotic, and bleeding events. Nevertheless, anticoagulation in patients with Impella devices can often be complicated due to unpredictable purge flow rates, pre-existing coagulopathy, or heparin allergies. In this setting, a sodium bicarbonate-based purge solution (25 mEq/1,000 ml 5% of dextrose) currently represents the reliable and approved alternative option (12). In addition, argatroban and bivalirudin have been employed in the purge solution too, appearing to be safe with no bleeding or thrombotic complications (13, 14).

Pharmacological management varies according to assistance strategy: in a recovery strategy the administration of Levosimendan is commonly used to facilitate weaning with increased success rate (15).

All these systems are approved in the United States (US) for adult use; despite this, randomized trials are still lacking. If the Bi-Pella approach presents encouraging outcomes in cardiogenic shock probably due to its capacity for biventricular unloading compared to ECMO, a recent meta-analysis (16) seems to show that ECMO coupled with an unloading device gives an advantage in terms of mortality reduction. Such recent strategy (the so-called ECPELLA) is very promising and seems to be associated with better outcomes. A recent multicenter study by Schrage (17) demonstrated that early active LV unloading with an Impella device is associated with a lower early mortality and a higher likelihood of successful weaning from ventilation, in the absence of more complications. Moreover, the interval between ECMO implantation and Impella initiation directly correlated with increased mortality and adverse outcome. The importance of an efficacious LV unloading is also confirmed by Isath (18), who shows that in the setting of ECPELLA, utilization of Impella 5.5 provides greater hemodynamic support with a lower risk of complications compared to Impella CP or 2.5. These findings suggest that in the optic of cardiac recovery, LV unloading is essential in order to completely “set aside” the heart. This translates in the need of ECMO patients' careful monitoring looking for the initial signs of pulmonary congestion (especially in case of very low residual cardiac contraction) that require the placement or the upgrading of ventricular unloading.

In patients under short-term support the question of the evaluation of systemic and cardiac recovery becomes crucial, in order to decide if weaning is possible or if a shifting towards a long-term assistance program or OHT is required. Different protocols of progressive decrease of the support are available and they vary among centers. In every step of weaning, systemic perfusion must be maintained (stable mean and systolic arterial pressure, appropriate cardiac index without increase in inotropic support) with normal levels of lactate and liver enzymes. In parallel, echocardiography must assess the recovery of LV function (LVEF > 20%–25%, LVOT VTI > 10 cm, Mitral TDSa > 10 cm/s) and of RV function (TAPSE ≥ 19 mm, RVEF ≥ 25%, low RA/PCWP, high PAPi), which must remain stable or improve during the flow reduction trial (19).

A perfect short-term BMCS should be simple to implant (ideally with a percutaneous technique) and to manage, two characteristics which render ECMO the current more suitable assistance when compared to a double Impella, which requires a complex anticoagulation protocol, a strict echocardiographic monitoring of the device position and the contemporary management of a right and of a left pump with different settings.

Nevertheless, ECMO presents the negative side of representing a machine which “supports the organs and not the heart”; we can describe it as a temporary tool to preserve the vital organs while waiting for cardiac recovery. This means that, for instance, it can be deleterious if used without a parallel device unloading the left ventricle in case of profound cardiac dysfunction. Obviously, hemolysis and long-term complications including platelet count fall and dysfunction and systemic inflammation are common persisting defects of all currently available short-term devices.

## Long-term biventricular devices

Several devices have been added to and taken off the market over the years: so far, available options are:

1. Syncardia CardioWest (SynCardia Systems, Inc., Tucson, AZ): TAH.
2. Berlin Heart EXCOR (Berlin Heart AG, Berlin, Germany): paracorporeal BiVAD.
3. LVAD + Right Ventricular Assist Device (RVAD): two HeartMate 3 (Abbott, Abbott Park, Illinois, USA): intracorporeal BiVADs.

All biventricular devices are approved for Bridge To Transplantation (BTT) treatment only (20).

Currently, the only device approved for adult use in the US is the Syncardia, while the Berlin Heart is reserved for pediatric use in the US and for patients of all ages in Europe. The third option of two HeartMates 3 (also in a TAH configuration—the so-called HeartMate 6—with both ventricles excised) is feasible but off-label.

Differently from paracorporeal or intracorporeal BiVADs, Syncardia TAH and HeartMate 6 require the excision of both ventricles, thus preventing the possibility of removing the devices in case of cardiac function recovery.

### TAH

#### Description

**Figure 4:**
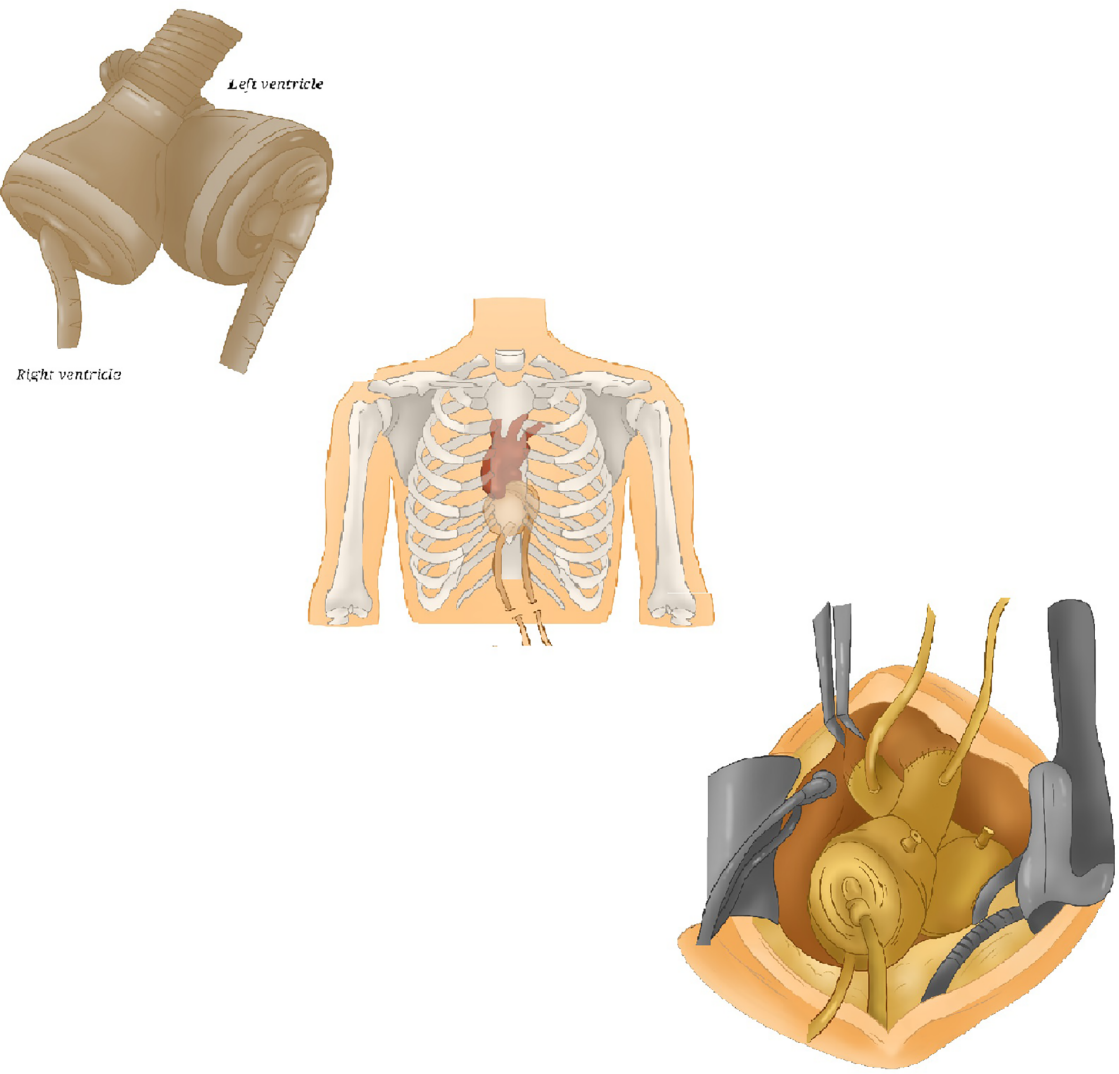
Syncardia TAH. Some schematic pictures of Syncardia, composed of two artificial ventricles, two outflow grafts and two drivelines. TAH, total artificial heart.

The Syncardia (Figure 4) is a pneumatically driven TAH consisting of two polyurethane ventricles with a stroke volume of 70 cc implanted for the first time in 1969 (21). Every chamber has two Medtronic-Hall mechanical disc valves providing unidirectional flow. Blood and air are separated by a four-layer polyurethane diaphragm that retracts during diastole and is displaced forward by compressed air during systole to expel blood. Even though the TAH can provide flows up to 10 L/min, it is usually used between 6 and 8 L/min.

#### Patients' selection

The TAH is indicated in biventricular HF patients eligible for OHT in INTERMACS class I–II–III with certain thoracic dimensions (Body Surface Area 1.7–2.5 m^2^ and a ≥10 cm distance between the tenth thoracic vertebra and the sternum) (22). Syncardia is for patients presenting with advanced right ventricular (RV) failure, contraindicating an LVAD: this is a key element of patients' global evaluation that is addressed by clinicians. Many scores have been developed to identify patients who may not need biventricular support, which is synonymous with more serious disease and worse outcomes (23). Another TAH indication is end-stage HF in patients with conditions that make them not suitable for LVAD support: hypertrophic, infiltrative or restrictive cardiomyopathies, anatomical conditions, intraventricular thrombi, cardiac cancers or ventricular tachyarrhythmias requiring complete heart excision (23).

#### Implantation technique

The principal steps are (22):

- Ventricular excision, pulmonary artery and aorta transection.
- Anastomosis of atrial connectors and of outflow conduits.

When the patient's thoracic size is on the borderline of the acceptable range, various technical tips have been suggested to find progressive device accommodation suitable for a correct hemodynamic status.

#### Management

The following parameters need to be set (22):

1. Left and right air activation pressures: they must allow aortic-pulmonary valve opening (usually 180 mmHg on the left side and 80/90 mmHg on the right).
2. Cardiac rate: between 120 and 130/min (due to the slightly decreased stroke volumes compared to normal physiology).
3. Systole length percentage: in contrast to a normal heart where diastole is longer than systole, a length of 50% length is usually optimal to achieve adapted filling and emptying.
4. Drivelines aspirating pressures (filling help).

The device automatically calculates filling volumes and bilateral cardiac output (with the right-sided one still 700–800 cc lower).

Syncardia works correctly when there is uncomplete filling followed by full emptying. Objectives of management consist of joining the theoretical cardiac output with a central venous pressure <15 mmHg (for avoiding hemorrhagic complications too).

#### Results

The first publication on TAH outcomes in BTT therapy was the multi-center American trial by Copeland (20), which showed an overall 1-year survival rate of 70% and a survival to OHT of 79%. In transplanted patients, at 1- and 5-years follow-up, survival reaches 86% and 64% respectively. More recently, other groups have produced comparable results: Torregrossa (24) declared that 72% of their 47 implanted patients underwent OHT, while Roussel (25) reported a mortality rate of 28% with an actuarial survival for transplanted TAH patients ranging from 90% at 1-year follow-up to 76% at 10 years. Pitié group's record (26) is less positive: in 90 consecutive implantations, the mortality rate was 39%; the remaining patients were transplanted after 3 months of support, showing an actuarial survival of 78% at 1-year and of 63% at 8-years follow-up. In 2012, Copeland (27) completed his initial population follow-up by publishing outcomes at 10-years: compared to the initial results, survival parameters go worse with 68% of patients receiving a transplant and a survival rate at 1–5–10 years after OHT decreasing to 78%–60%–41% respectively.

Multivariate analysis reveals that risk factors for death with TAH support are age, smoking and preoperative mechanical ventilation, while a prothrombin time of ≥16 s and smoking are associated with a higher mortality rate after transplantation (26, 28).

#### Complications

The main complications following Syncardia implantation are stroke, infections, thromboembolic events, renal failure and chronic multi-factorial anemia. The largest TAH trial (22) reports a major stroke rate of 8%, an infection rate of 63% (mostly affecting the lungs and the urinary tract), a significant hemorrhagic episode rate of 43% and a driveline infection rate of 27% (19). Although deep mediastinal infection is relatively rare (3%–15%) (28), almost two thirds are life-threatening.

Severe postoperative renal impairment is quite frequent (10%) and is due to a sudden fall in Brain Natriuretic Peptide levels secondary to ventricular excision which may interrupt its physiologic compensatory role (29). Device failure rate is approximately 10% (24).

#### Syncardia 50 cc

Until May 2016, about 1,600 TAHs had been implanted worldwide, with only 12% of these in women and less than 5% in children due to problems with thoracic anatomy (30). Recently, a smaller 50 cc Syncardia has been introduced for patients with a BSA ≤ 1.2 m^2^. This device is comparable to the 70 cc TAH, apart from the 29% reduction in its size. It has two 50 cc ventricles and 25 mm-inflow and 23 mm-outflow valves (Table 3).

**Table 3:** Syncardia 70 cc and 50 cc technical features.

Feature	Syncardia 70 cc	Syncardia 50 cc
Weight (g)	240	200
Left ventricle dimensions (mm)	74*80	77*66
Right ventricle dimensions (mm)	74*90	75*80
Inflow valve size (mm)	27	25
Outflow valve size (mm)	25	23
Displaced volume (ml)	400	250
Maximal stroke volume (ml)	70	50
Maximal flow (L/min)	10.5	7.5
Purge system	Yes	No

Preliminary results described by the 50 cc TAH Clinical Study (30) show a positive outcome in 50% of patients.

### Paracorporeal devices

#### Berlin heart EXCOR

**Figure 5:**
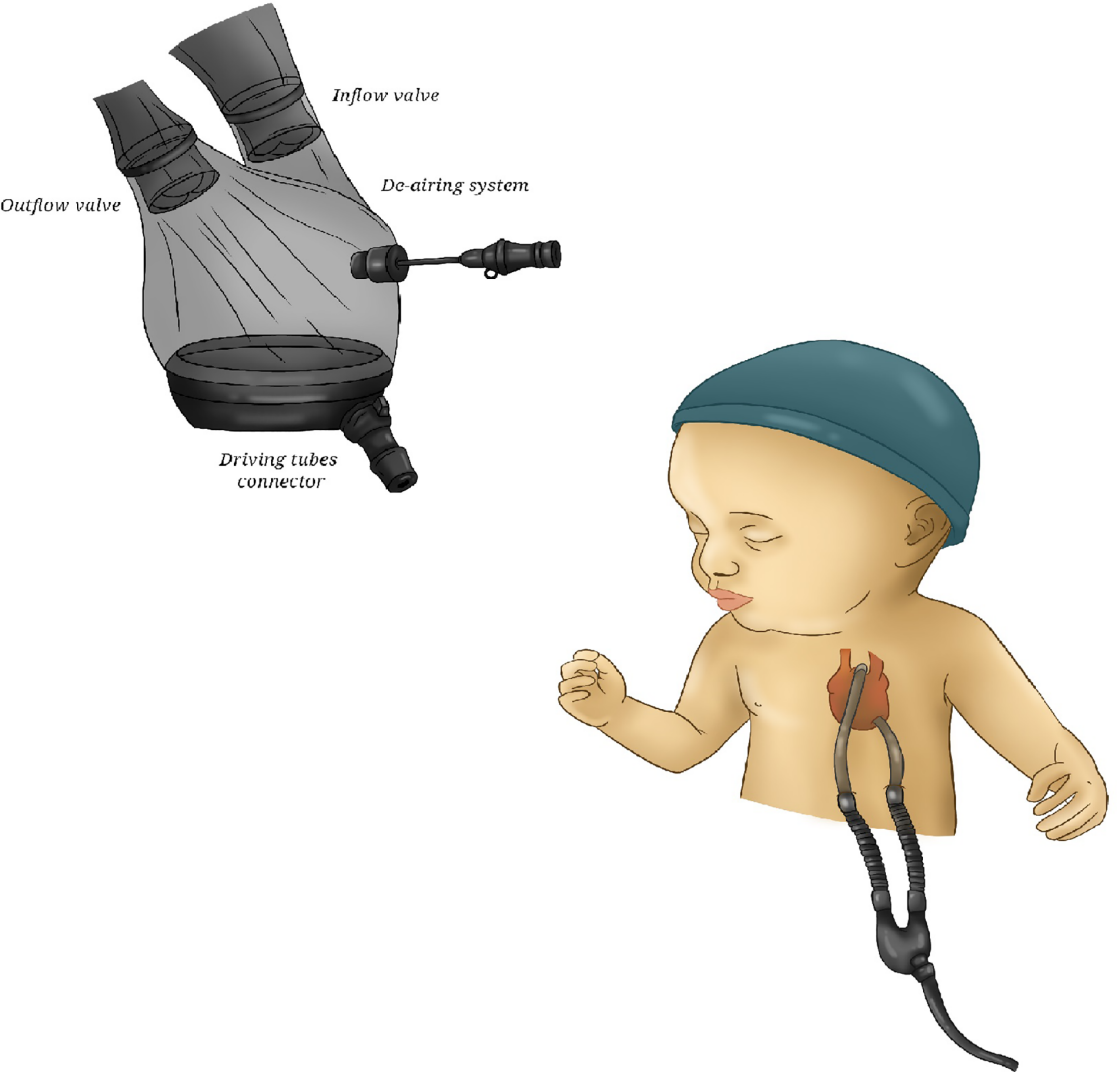
Berlin Heart EXCOR. A picture of Berlin Heart, showing its transparent housing and bileaflet valves.

This pneumatic-pulsatile mono- or biventricular paracorporeal device, available for short-, mid-and long-term support, derives from the Thoratec (Thoratec Corporation, Pleasanton, CA, USA) technology (31, 32) (Figure 5). It consists of an external pump with inflow and outflow cannulas that traverse the skin and connect the pump with the heart and the great vessels.

The pump chamber has a flexible three-layer polyurethane membrane separating the air side connected to the driving unit and the blood one connected via the cannulae to the patient's circulation. The blood is propelled by cyclically changing the internal volume of the compression chamber through a pneumatically-driven expansion of the membrane, directing the flow with one-way valves. The energy source is an external smaller and portable compressor. The transparent housing allows for immediate detection of thrombi formation and malfunctions. By that, pump exchange can be performed easy without the need for lysis therapy. To avoid friction, a graphite lubricant powder lies between the layers. The blood-contacting surfaces of the pump are coated with heparin (Carmeda®). It is available in several sizes with different pump volumes (10, 15, 25, 30, 50, 60, 80 ml) and cannula sizes in order to support patients of all ages. Initially, adult sizes were furnished with mono-disc mechanical prostheses; since August 2004 they have been replaced by heparinized bileaflet valves. This evolution has resulted in a reduction of pump noise and in simpler preparation and de-airing processes. The driver-console, provided with rechargeable batteries, is portable.

#### Implantation technique

Step-by-step EXCOR cannulation process consist of: left ventricle apex, right atrium, pulmonary artery trunk, and ascending aorta (32). Direct cannulation of the left atrium is preferred in patients affected by restrictive or hypertrophic cardiomyopathy.

#### Management

Basing on BSA and theoretical cardiac index, patient's adapted pump size is chosen. To obtain the required flow, an appropriate rate on the driving unit is set (usually 80–100/min). Systolic and diastolic pressures must be set and dynamically adapted to change in pre- and afterload for allowing a complete deflection of the blood pump membrane. A systole percentage set between 30% and 40% should allow a complete filling and emptying of the pump. Management goal are a central venous pressure around 10 mmHg and mean arterial pressure around 70–80 mmHg.

#### Results

A recent trial (28) on Berlin Heart shows a global survival rate of 75% at 5-years follow-up. Complications are not negligible: a 33% rate of re-interventions for major bleeding, 20% rate of chamber thrombosis or partial membrane rupture, 25% rate of strokes and 40% rate of infections. Conversely, a German prospective of 12 patients (33) highlights encouraging results, describing a 1-year survival of 92% and an incidence of thromboembolic, infectious and thoracic bleeding complications of 25% in each category.

### Continuous-flow BiVADs

#### Introduction

Continuous-flow devices are efficacious and safe for the left heart and significantly extended patients' mean survival by up to 7.1 years after implantation (34). Technology evolved from axial pumps to miniaturized centrifugal currently used devices (HeartMate 3).

#### HeartMate 3

**Figure 6:**
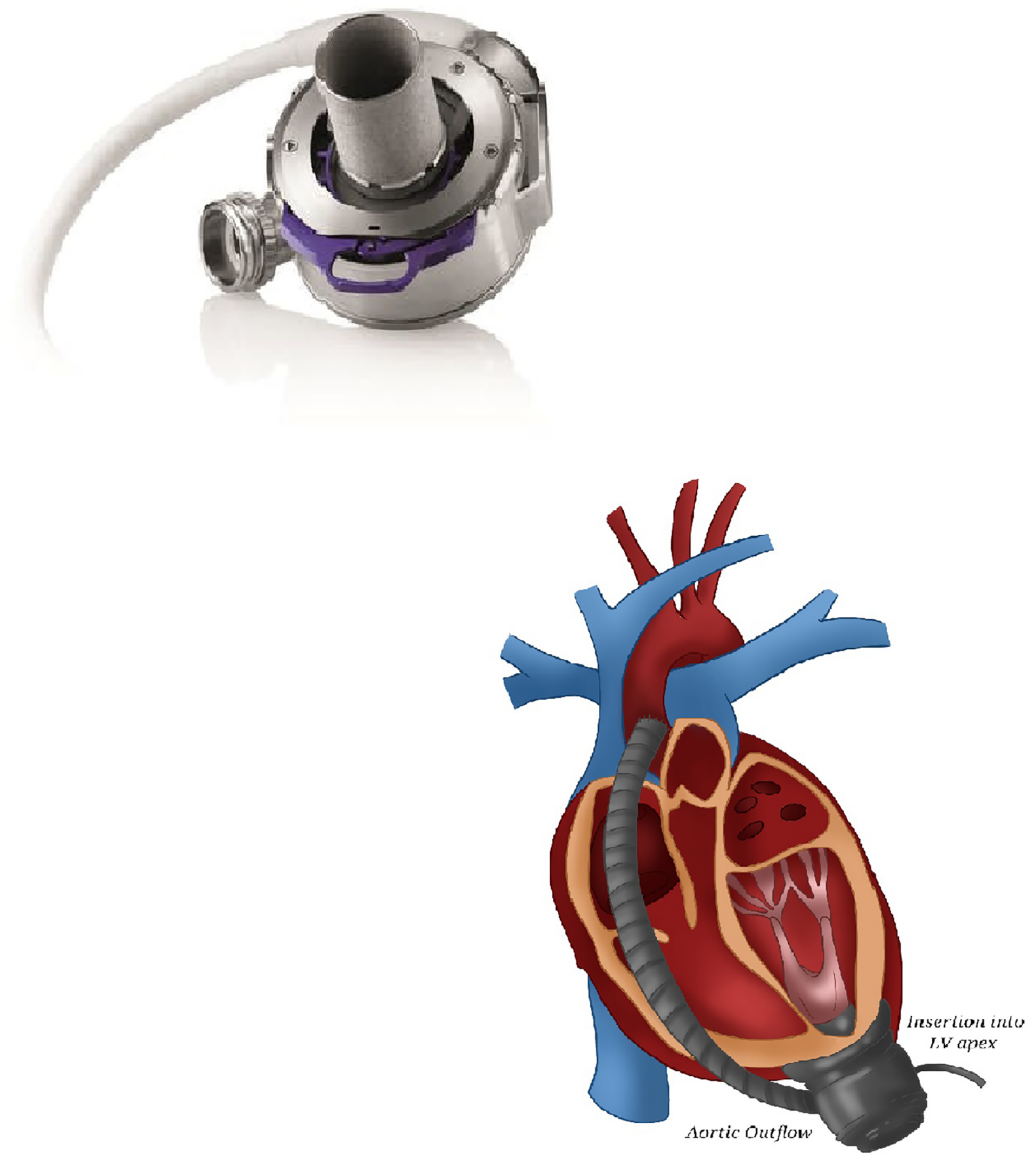
HeartMate 3. A picture of HeartMate 3 and of its intrathoracic placement.

Ancient LVADs were burdened by pump thrombosis and strokes which encouraged the industries to improve the hemocompatibility of these devices (Figure 6). The most recent generation of the HeartMate 3 pump is an intra-pericardial centrifugal assistance device provided with full magnetic levitation technology. This uses a frictionless rotor with an intrinsic fixed pulsatility generated by a sequential increase and reduction of rotor speed every 2 s. The device is composed of an inflow cannula, a pump housing, electronic controllers, an outflow graft and a percutaneous driveline. In contrast to its predecessors, contactless rotation and larger spaces in the blood pathways allow a reduction in shear stress and a fast change in rotation speed to reproduce a pulsatile flow. This prevents hematic stasis and consequently hemolysis (34).

#### Implantation technique

Classic LVAD requires placement of the inflow cannula in the left ventricular apex and of the outflow cannula in the ascending aorta (Figure 6).

**Figure 7:**
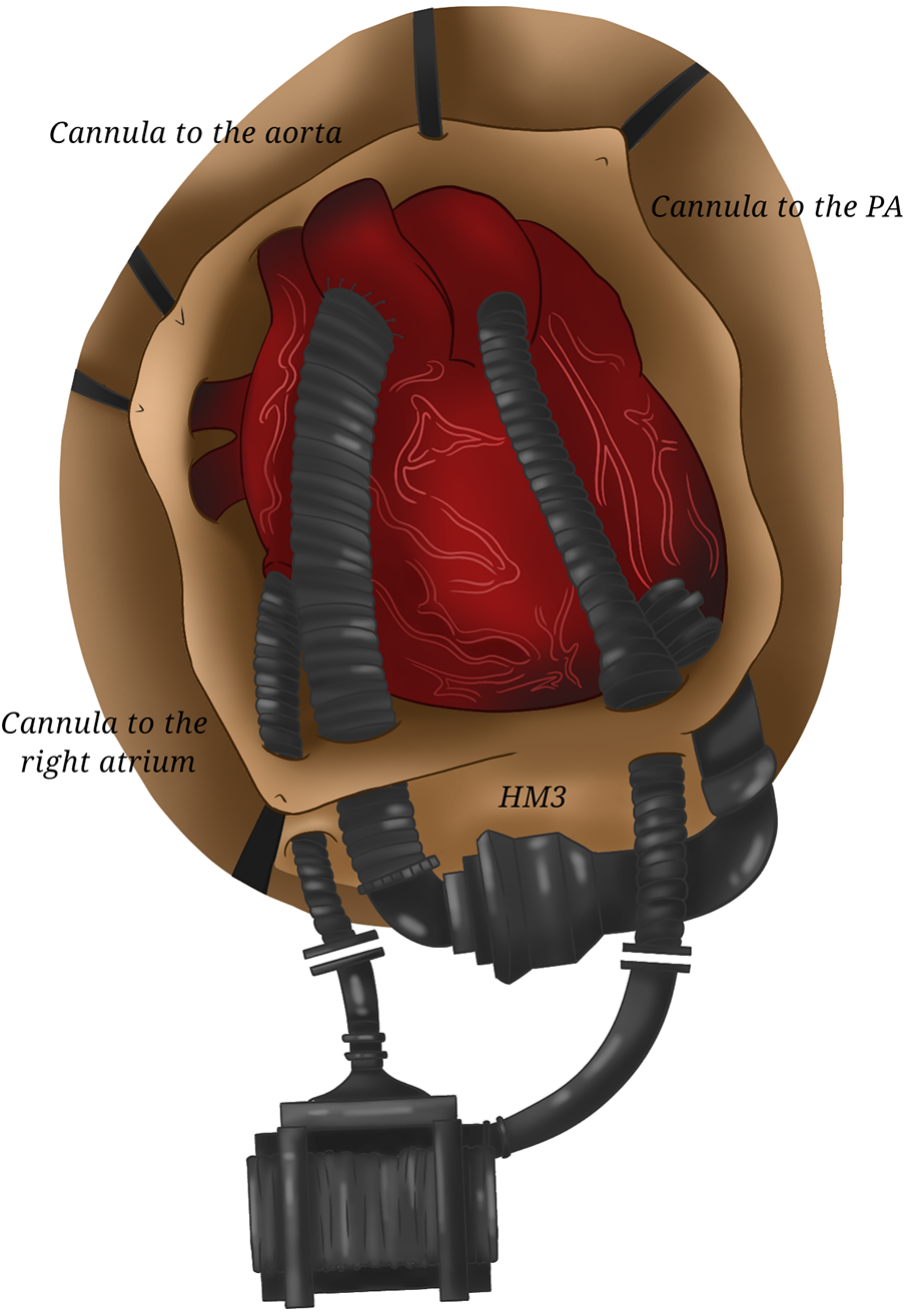
Two HeartMate 3. The coupling of two HeartMate 3 requiring the cannulation of the right atrium and of the pulmonary artery (for the right device) and of the left ventricular apex and of the aorta (for the left one) allows total cardiac assistance.

In off-label use of two continuous-flow pumps for total assistance (Figure 7), three problems arise:

1. LVADs are designed for systemic circulation: the right outflow graft diameter is surgically reduced to increase right afterload for avoiding pulmonary edema (35).
2. Inflow cannulas are too long for the average RV and need shortening.
3. The optimal anatomic placement (right ventricle vs. right atrial) for the right pump allowing for the lowest risk of pump thrombosis (36) is still controversial.

#### BiVADs in TAH configuration (HeartMate 6)

An alternative surgical technique consists of biventricular excision achieving an arrangement resembling the Syncardia-TAH with the sewing rings sutured to the atrial cuffs; although such a strategy should avoid problems related to correct right device orientation, it still remains off-label and requires further validation (37).

#### Management

Rotations per minutes are set in order to obtain the theoretical left cardiac output (which is automatically calculated by the system using several parameters including speed and patient's hematocrit) and a right one 500–1,000 cc lower with classical left speed being between 5,000 and 5,500 rpm.

#### Results

Available trials on continuous-flow BiVADs are based on small cohorts: in 13 implanted patients (50% in a ventricular configuration), Shehab (38) reported a 54% survival after 269-days mean support, with a significant rate of pump thrombosis, infection and hemorrhagic complications. A meta-analysis on 56 patients in 13 trials (39) confirms a better survival with atrial cannulation (91% vs. 66% at 1-year follow-up) without major device-related adverse events.

The review by Farag (40) summarizes the performance of a double ventricular assist device strategy in biventricular support (only 3 studies with two HeartMates 3): 30-days mean survival reaches 90% with a fall to 58.5% at 1-year follow-up. Pump thrombosis (mostly of the right machine) occurred in 31% of patients with a pump change needed in 9%.

So far, more than 400 continuous-flow BiVADs have been implanted worldwide.

## Biventricular strategies outcome comparison

Several papers have compared the results of each approach: a recent meta-analysis (41) evaluated TAH and two continuous-flow BiVAD devices at 120 days follow-up, highlighting a similar mortality rate (36% vs. 26% respectively) but a better quality of life for intracorporeal devices with more patients discharged home despite a longer duration of support. Rates of hemorrhagic complications and infections were comparable. Levin (42) drew the same conclusion and confirmed that monoventricular assistance is associated with a better survival rate (92.7%).

Interestingly, a retrospective study (43) on a previous implantation period (2004–2012) observed an increased mortality for Syncardia, suggesting a technical improvement in the subsequent years. Conversely, the “Groupe de Réflexion sur l’Assistance Mécanique” describes a similar mortality rate before (75% at 30-days and 57% at 180-days follow-up) and after OHT (81% at 1-month and 64% at 5-years follow-up) in all BiVADs types and a trend towards an increased survival with >90 days of Syncardia support, probably due to a lower incidence of strokes (44).

## Common BMCS pharmacological treatment

All BMCS require the coupling of anticoagulation and antiplatelet drugs therapies with variable protocols. As for short-term devices, the degree of anticoagulation needs to be adapted to every patient, especially in the first hours after the implantation of the device, considering the individual perioperative bleeding state and risk.

Usually, the standard of care is represented by an initial treatment with unfractioned heparin, followed by vitamin K antagonists such as warfarin with the adjunct of acetylsalicylic acid for long-term maintenance. Nevertheless, bivalirudin is gaining space due to its short half-life, lack of dependence on antithrombin, and more stable pharmacokinetics; thus, at present, its use is not limited to situations wherein heparin is ineffective or contraindicated. Especially in children assisted with continuous flow devices or Berlin Heart, outcomes with direct thrombin inhibitors (bivalirudin or argatroban) are encouraging with lower major bleeding and stroke event rate than that reported with unfractioned heparin (45).

Concerning long-term anticoagulation, preliminary evidence suggests that new direct oral anticoagulants, which directly inhibit Factor Xa (rivaroxaban, edoxaban and apixaban) or Factor IIa/thrombin (dabigatran), may be a valuable alternative to warfarin in adults patients assisted with continuous-flow LVAD, but further investigations are needed (46).

No other specific treatments are needed when biventricular dysfunction is judged irreversible. Otherwise, in case of possible partial or complete recovery (typically in peripartum cardiomyopathy and myocarditis) and if biventricular implantation is not in a TAH configuration, all drugs employed in an acute HF setting, such as inotropes and levosimendan, and in the treatment of chronic systolic heart failure, may be administered. More in detail, neurohormonal inhibitors (angiotensin-converting enzyme (ACE) inhibitors, angiotensin receptor blockers (ARBs), aldosterone antagonists and beta blockers) could facilitate recovery (47) and significantly reduce the risk of mortality in VAD patients (48).

Positive hemodynamic effects have also been demonstrated with other vasoactive substances, including exogenous natriuretic peptides (nesiritide, ularitide), the endothelin-1 antagonist tezosentan, cinaciguat (an activator of soluble guanylate cyclase) and serelaxin, but their role in acute decompensated HF remains uncertain (49).

## HF patients' practical management: how to select the right device for the right patient

In acute cardiogenic shock requiring assistance, no time is available for speculation and ECMO is the preferred MCS for its simple implantation. Once hemodynamic situation is stabilized, patient's evaluation and estimation of MCS perspective are mandatory. Clinicians must answer to the following questions:

1. Prevalent mono- or biventricular failure?
2. Chance of recovery of every ventricle.
3. Pulmonary status.
4. Eligibility to OHT.

Different scenarios may arise:

- Need for longer short-term MCS to allow recovery or bridge-to-decision: shifting vs. CentriMag or biventricular percutaneous assistance combination is conceivable.
- Satisfactory RV function: consider LVAD implantation or OHT.
- In case of no chance of recovery and no contraindications, OHT remains the best option.
- If biventricular failure persists along with temporary contraindications to OHT, long-term MCS are required. Syncardia still remains the gold standard due to its diffusion and proved efficacy; Berlin Heart and, in selected cases, an off-label strategy employing two continuous-flow BiVADs, are two valuable alternatives gaining momentum, especially if more time is needed to evaluate recovery chance.

## The challenge of hospital discharge under BMCS

According to the INTERMACS Registry, 24% of patients implanted with a Syncardia were discharged home with their device after 1.6 months, with this number increasing over time. Even if TAHs improve quality of life for most of the implanted patients, rehabilitation and hospital discharge remain very challenging for patients and their care providers. A 6-kg driver that permits hospital discharge is now available to patients waiting for OHT and supported with the Syncardia. For years, one of the major disadvantages of the use of a TAH was the necessity of keeping the patient in-hospital while waiting for transplantation. Clinical experience with the new small TAH driver has allowed a few centers to discharge patients home (50).

Concerning Berlin Heart Excor, the possibilities of patients' hospital discharge seem more encouraging: adult patients requiring a blood pump size of 60 and 80 ml can be discharged with the EXCOR R Mobil by Berlin Heart GmbH. So far, the only other option for patients requiring smaller blood pump sizes is in-hospital treatment with the stationary IKUS driving unit (Berlin Heart GmbH) that can be used for all EXCOR R blood pumps. Unfortunately, the IKUS has a battery life of only 30 min and thus considerably limits patients' mobility. Focusing on this particular patient group, Berlin Heart GmbH has recently developed a novel electro-pneumatic mobile drive unit called EXCOR R Active, that has been especially designed for the operation of the paracorporeal system EXCOR R VAD and can be used for either univentricular assist or for biventricular assist. The use of the EXCOR R VAD in combination with EXCOR R Active as its driving unit has already been proven to be feasible for internal and external usage within the setting of professional health institutions. Unfortunately, this system, which has a guaranteed battery life of approximately 12 h, has not yet been approved for use in a home healthcare environment (51).

The experience with two continuous-flow BiVADs is currently too limited for drawing any conclusion about its capability to allow a simpler hospital discharge compared to TAH or Berlin Heart. If, on the one hand, the portable console unit is quite small and light, on the other hand, the patient is obliged to carry two consoles with two distinct settings, making it a little more difficult to achieve an independent and satisfying quality of life.

## Future perspectives

### How should be the perfect long-term BMCS?

In patients needing long-term biventricular support, the available options are far from optimal despite acceptable outcomes that are independent of device choice. Syncardia is bulky and noisy, leading to a limited quality of life. The Berlin Heart in biventricular configuration is not approved for adults use in the US; anyway, it has four mechanical valves and two drivelines, although its strong points are that it is less heavy, less loud and is suitable for every patient. With regard to continuous-flow BiVADs, the lack of a right-heart specific device is problematic: an *ad hoc* machine would decrease the number of mistakes and reduce the risk of pump thrombosis.

The conclusion is that the ideal long-term biventricular support is far to be found. Engineers and researchers are strongly working on this field, trying to ameliorate all the current defects of the available machines. The most important objective would be to have a device which allows fast patient's hospital discharge, assuring a satisfying and pseudo-normal quality of life. This means that it should be noiseless, with a little and portable controller or, even better, equipped with a transcutaneous charge, a feature which could also greatly reduce the rate of one of the most frequent complications of BMCS represented by drivelines infections. From a surgical point of view, the implantation technique should be simple and standardized ensuring a safe incoming transplantation. An ideal device should also be removable in case of cardiac recovery.

Another fundamental issue that needs to be addressed is hemocompatibility: pump thrombosis and its related adverse events (strokes, systemic embolisms) remains the Achilles heel of BMCS despite aggressive antiacoagulant and antiplatelet therapies which may furthermore cause gastrointestinal or cerebral bleeding. Consequently, in order to mitigate the phenomena of thrombosis, future MCS devices must focus their attention on improving the design of pump geometry, the materials, and the blood-contacting surfaces (52). In this optic, a new pumping concept—the so-called progressive wave pump- is under study: it consists in the interaction of an elastic membrane actuated by forced excitation with a surrounding fluid and the pump housing which is expected to reduce blood trauma, to increase hydraulic performance, and perhaps to require lower levels of anticoagulation (53).

Some promising trials are underway with the BIVACOR BV System (BIVACOR PTY Ltd, Brisbane, Australia).

An alternative line of development concerns CARMAT-TAH device (Vélizy-Villacoublay, France).

### BIVACOR

BIVACOR is a unique pump developed by BIVACOR Inc. (Houston, TX, USA). It consists of a single chamber, separated by a dual sided rotor designed to provide centrifugal flow to both the systemic and the pulmonary circulation. There are four ports of attachment to the native circulatory system—an aortic port, a pulmonary artery port and two atrial ports for the left and right atrium, respectively. The rotors have different outer diameters to produce the pressure required of the systemic and pulmonary systems at a common rotational speed. BIVACOR performance has been tested in a simulated pulsatile loop, reproducing an end-stage HF situation: it reestablished a correct flow of 5 L/min starting from 2 L/min (54).

This device is expected to remove many complications of current available BMCS: first, the setting is simpler, since there is no need to separately adjust the parameters of a systemic and of a pulmonary assistance thanks to its ability to alter and adapt the left and right outflow following the changes in vascular resistance or contingent abnormalities occurrence; second, it can operate successfully alongside a functioning RV in case of recovery; third, the double-side impeller configuration of the BIVACOR and its modulable pulsatile flow reduce the potential for thrombus formation by eliminating areas of low flow or stagnation often found beneath single-side centrifugal blood pump impellers. Last, this TAH presents a relatively small size which enables intrathoracic placement: a virtual fitting model showed that it fit well within the chest cavity of all tested patients (55).

Nevertheless, similarly to Syncardia, it requires cardiac excision, resulting in the impossibility of device explant if a sufficient biventricular contraction is restored. In addition, due to the coupled nature of the right and left impeller, the capacity of accommodation of the respective outflow may be impaired in case of contemporary changes in systemic and pulmonary vascular resistance (e.g., systemic vasodilation along with pulmonary hypertension). In this circumstance, medical management becomes mandatory. Finally, differently from Syncardia, no mechanical valves are included in the device, but at least a low level of anticoagulation is still needed.

### CARMAT

**Figure 8:**
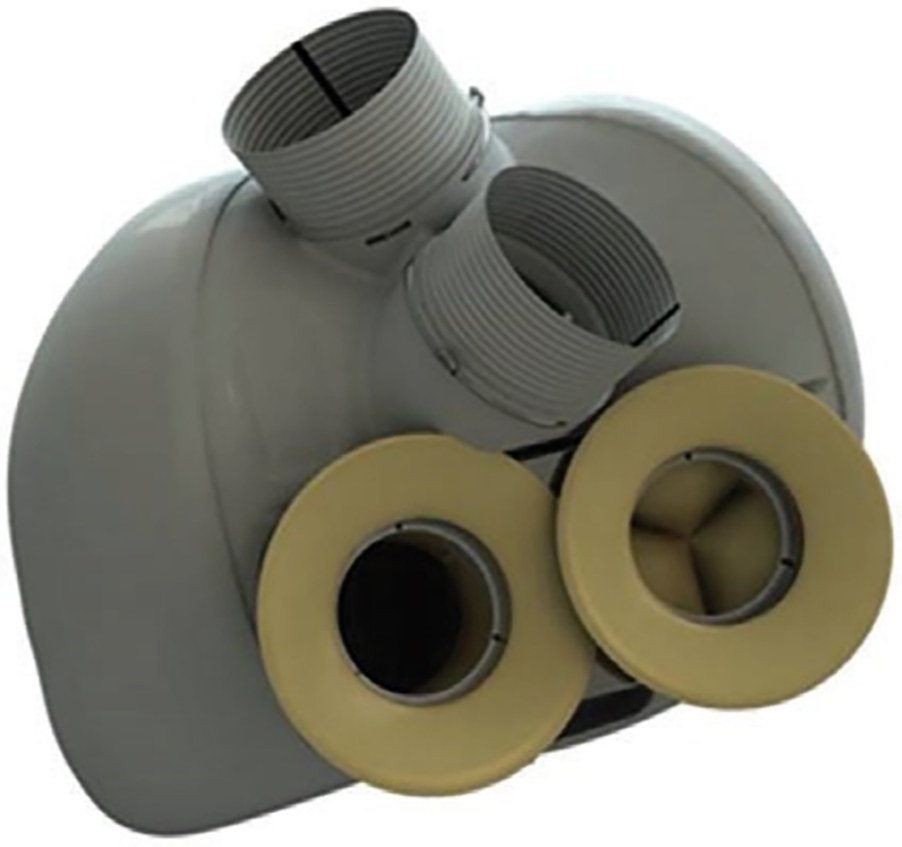
CARMAT. CARMAT external aspect showing the bag surrounding the device, the atrial sewing collars, and the outflow grafts.

CARMAT's history began in Paris in 1993 (56). This TAH (Figure 8) contains two chambers, each of which is separated by a membrane into a blood and a fluid compartment. The viscous fluid, set in motion by two electro-hydraulic pumps, actuates the membranes, producing pulsatile flow through four biological valves. Pressure sensors that drive the system and adapt to the patient's needs are embedded in the prosthesis. The CARMAT is connected to the atria with bioprosthetic suture flanges and requires, as with Syncardia, the excision of both ventricles, leaving no chance of cardiac recovery; Dacron outlet conduits are sutured onto the aorta and pulmonary artery. A percutaneous driveline connected to a console provides power. The surface of the blood-contact membrane is a bioprosthetic material-processed bovine pericardial tissue designed to improve hemocompatibility. The internal electro-hydraulic actuation of the membranes eliminates the need for an external actuator and produces no noise. The device uses a control algorithm that responds to changes in preload and afterload, resulting in blood flow ranges from 2 to 9 L/min. While this might reduce the risk of user-interface errors, failing components cannot be replaced without device explantation. The company provides a virtual anatomical fit tool to select patients with enough thoracic space (estimated at nearly 86% of male and 14% of female patients): for widespread use, a miniaturized version is necessary.

After a first implantation in France in 2013 on a 76-years-old patient, CARMAT received Food and Drug Administration approval in 2020 for the recruitment of 10 patients eligible for OHT in the US and then, in February 2021, for initiating a new American feasibility trial. CE mark was obtained on December 2021 for a BTT implantation.

Since the PIVOTAL study in 2016, CARMAT's analysis of the first 10 patients demonstrated that 70% survived or were successfully transplanted. The absence of hemolysis or major complications was reassuring, with lighter anticoagulation required compared to Syncardia or LVADs.

## Conclusions

**Figure 9:**
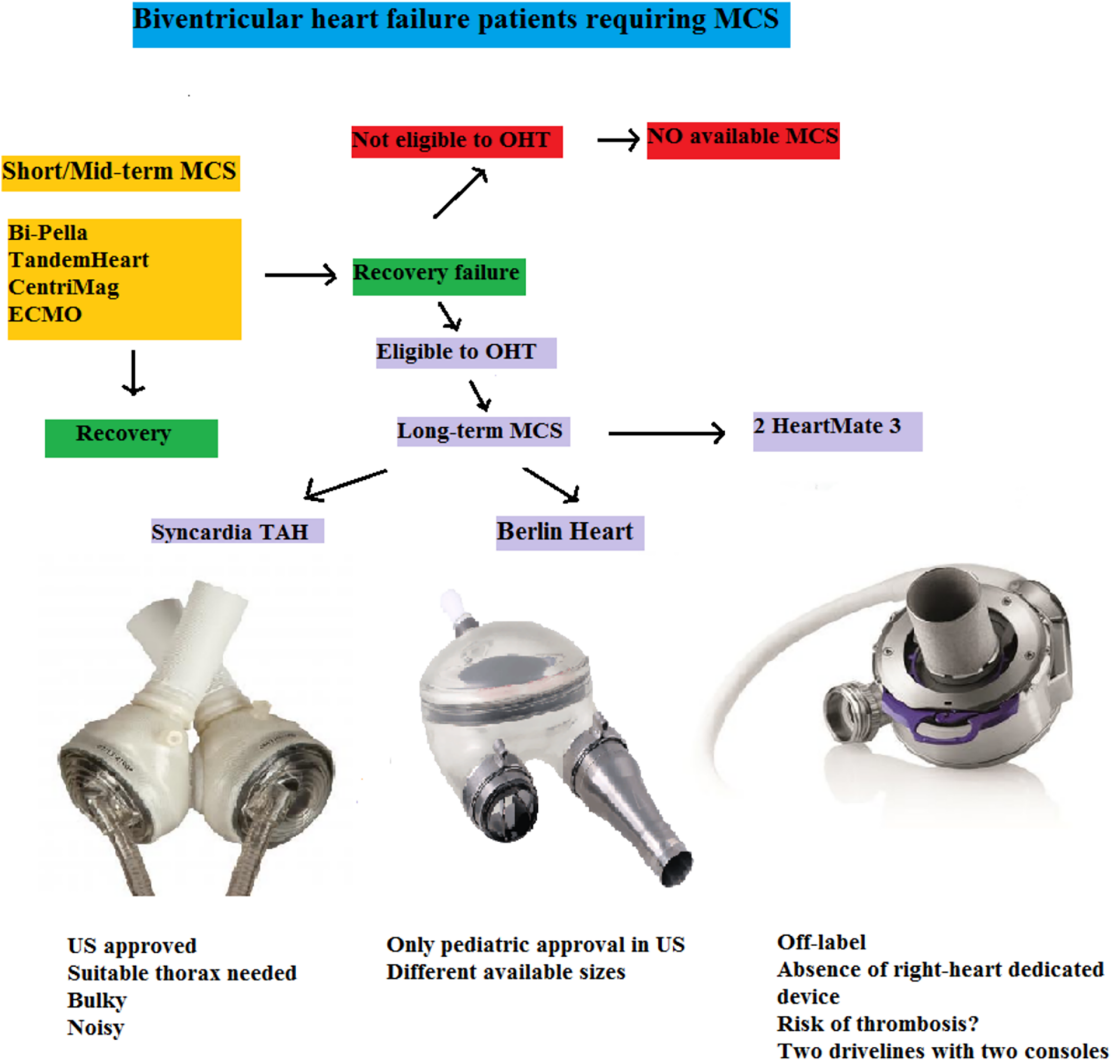
Flow-chart of biventricular heart failure patients requiring MCS. Short/Mid-term MCS may lead to recovery or bridge to long-term MCS. In a destination-therapy setting, no form of mechanical assistance is currently approved. For BTT patients, two adult devices (one off-label in the US) and a pediatric one are available, each presenting pros and cons highlighting the lack of an optimal solution. ECMO, extracorporeal membrane oxygenator; MCS, mechanical circulatory support; OHT, orthotopic heart transplantation; TAH, total artificial heart; US, United States.

The ideal BMCS does not yet exist. Available tools (TAH or BiVADs) are exclusively indicated in a BTT therapeutic strategy (Figure 9) and ensure a survival rate of about 70%, confirming the gravity of such a clinical condition. Moreover, they are associated with a significant incidence of major complications (infective, thromboembolic, hemorrhagic) and greatly limit patients' quality of life because of their encumbrance and noise. Aside from this, no solution for destination therapy is available. Several clinical and pre-clinical trials concerning new devices that can potentially address the weaknesses in BMCS are underway, with encouraging preliminary results.
